# Associations between the gut microbiota and host responses to high altitude

**DOI:** 10.1152/ajpgi.00253.2018

**Published:** 2018-09-13

**Authors:** J. Philip Karl, Claire E. Berryman, Andrew J. Young, Patrick N. Radcliffe, Tobyn A. Branck, Ida G. Pantoja-Feliciano, Jennifer C. Rood, Stefan M. Pasiakos

**Affiliations:** ^1^Military Nutrition Division, United States Army Research Institute of Environmental Medicine, Natick, Massachusetts; ^2^Oak Ridge Institute for Science and Education, Belcamp, Maryland; ^3^Soldier Performance Optimization Directorate, Natick Soldier Research, Development and Engineering Center, Natick, Massachusetts; ^4^Pennington Biomedical Research Center, Baton Rouge, Louisiana

**Keywords:** gut barrier, hypoxia, macronutrient, microbiome, weight loss

## Abstract

Hypobaric hypoxia and dietary protein and fat intakes have been independently associated with an altered gastrointestinal (GI) environment and gut microbiota, but little is known regarding host-gut microbiota interactions at high altitude (HA) and the impact of diet macronutrient composition. This study aimed to determine the effect of dietary protein:fat ratio manipulation on the gut microbiota and GI barrier function during weight loss at high altitude (HA) and to identify associations between the gut microbiota and host responses to HA. Following sea-level (SL) testing, 17 healthy males were transported to HA (4,300 m) and randomly assigned to consume provided standard protein (SP; 1.1 g·kg^−1^·day^−1^, 39% fat) or higher protein (HP; 2.1 g·kg^−1^·day^−1^, 23% fat) carbohydrate-matched hypocaloric diets for 22 days. Fecal microbiota composition and metabolites, GI barrier function, GI symptoms, and acute mountain sickness (AMS) severity were measured. Macronutrient intake did not impact fecal microbiota composition, had only transient effects on microbiota metabolites, and had no effect on increases in small intestinal permeability, GI symptoms, and inflammation observed at HA. AMS severity was also unaffected by diet but in exploratory analyses was associated with higher SL-relative abundance of *Prevotella*, a known driver of interindividual variability in human gut microbiota composition, and greater microbiota diversity after AMS onset. Findings suggest that the gut microbiota may contribute to variability in host responses to HA independent of the dietary protein:fat ratio but should be considered preliminary and hypothesis generating due to the small sample size and exploratory nature of analyses associating the fecal microbiota and host responses to HA.

**NEW & NOTEWORTHY** This study is the first to examine interactions among diet, the gut microbiota, and host responses to weight loss at high altitude (HA). Observed associations among the gut microbiota, weight loss at HA, and acute mountain sickness provide evidence that the microbiota may contribute to variability in host responses to HA. In contrast, dietary protein:fat ratio had only minimal, transient effects on gut microbiota composition and bacterial metabolites which were likely not of clinical consequence.

## INTRODUCTION

The human host and its gut microbiota coexist in a dynamic mutualistic relationship, with the host providing a favorable environment for microbes that, in turn, modulate gastrointestinal (GI) health, GI barrier integrity, immunity, and inflammation (13, 35, 36). However, this relationship can be perturbed by exposure to environmental and physiologic stressors that alter the GI environment, the gut microbiota, or both (38, 49). Consequences can include degradation of GI barrier integrity leading to GI distress, translocation of bacterial antigens (e.g., LPS) from the gut lumen into circulation, systemic inflammation, and increased susceptibility to illness and infection (73, 74).

Hypobaric hypoxia is a physiologic stressor which characterizes high-altitude (HA; >2,500-m elevation) environments. Exposure to hypobaric hypoxia is associated with increased inflammation (34), increased risk of illness and infection (41, 54), and increased acute mountain sickness (AMS) (7, 55), a constellation of symptoms that includes several GI maladies (4). Rodent studies suggest that host-gut microbiota dynamics could contribute to these responses, demonstrating that exposure to hypobaric hypoxia increases GI inflammation, oxidative stress, and GI permeability concomitant to changes in gut microbiota composition and activity (1, 82, 86, 88). Although few human studies have attempted to corroborate those findings, transient increases in GI permeability (21) and increases in the abundance of proinflammatory gut bacteria (2, 42) in association with inflammation (42) have been reported during mountaineering expeditions. These observations collectively suggest that the gut microbiota may both be affected by and contribute to host responses at HA, but greater characterization of these relationships is needed.

Gut microbiota composition and activity are also modulated by dietary macronutrient intake (19, 28, 57, 80). It is well established that carbohydrate fermentation by the gut microbiota promotes the growth of beneficial bacteria and production of the short-chain fatty acids (SCFAs) acetate, propionate, and butyrate (48, 75), whereas amino acid fermentation produces multiple byproducts including SCFAs, branched short-chain fatty acids (BCFAs; e.g., isovalerate and isobutyrate), and ammonia (48, 75). While SCFAs, and butyrate in particular, are beneficial to GI health, several amino acid fermentation metabolites (e.g., ammonia) may harm the GI barrier (75), suggesting that protein fermentation could be deleterious to GI health (83). At sea level (SL), consuming higher protein diets that are also high in fat (>40% total energy), low in carbohydrate (<20% total energy), and low in fiber has been associated with reductions in beneficial gut bacteria and fecal SCFAs and increased fecal concentrations of amino acid fermentation metabolites (12, 19, 24, 25, 57, 65). When macronutrient intakes have been more consistent with dietary recommendations and fiber intakes have been matched between groups, higher protein diets have been shown to increase fecal concentrations of amino acid metabolites without impacting fecal microbiota composition (5, 79) or markers of GI health (5, 8, 79). Thus protein fermentation does not appear to acutely degrade GI barrier function when fiber intake is controlled. However, none of these studies were conducted in environments such as HA that may render the GI barrier more sensitive to gut microbes and their metabolites.

Recent interest in studying higher protein, moderate carbohydrate diets at HA (59) has been driven by the knowledge that unintentional fat-free mass loss is common during HA sojourn (33, 78), and that higher protein diets preserve fat-free mass during weight loss at SL (60, 81). However, the effects of these diets on host-gut microbiota dynamics at HA are unknown. This study aimed to both address that gap and the need for greater characterization of host-gut microbiota dynamics at HA by *1*) determining the effect of altering the dietary protein:fat ratio on GI barrier function, GI symptoms, and gut microbiota composition and metabolites during weight loss at HA; and *2*) identifying associations among the gut microbiota, weight loss at HA, and the host response to HA as measured by AMS severity.

## METHODS

### 

#### Participants and study design.

The analyses reported herein were included as secondary objectives in a randomized, controlled feeding study designed to examine the efficacy of a higher protein diet for preserving fat-free mass during HA sojourn (9). Participants were 17 healthy, unacclimatized, physically active men. Although study enrollment was open to women, none volunteered. Participants had no GI abnormalities or disorders, had not taken oral antibiotics or had a colonoscopy in the previous 3 mo, and did not regularly use laxatives, stool softeners, or antidiarrheal medications. The study was approved by the Institutional Review Board at the U.S. Army Research Institute of Environmental Medicine (Natick, MA) and conducted May–Aug 2016. Investigators adhered to the policies for the protection of human participants as prescribed by Army Regulation 70-25, and the research was conducted in adherence with the provisions of 32 CFR Part 219. (The trial is registered on https://clinicaltrials.gov/ as NCT02731066.)

Study methods have been previously reported in detail (9). Briefly, the study was a randomized, controlled trial consisting of two phases conducted over 43 consecutive days. During the 21-day first phase (SL), participants resided at SL, consumed a self-selected weight-maintaining diet, maintained habitual exercise routines, and were free living but visited the laboratory daily. On SL *day 21*, participants were flown from Boston, MA to Denver, CO, where they were placed on supplemental oxygen until being driven to the summit of Pike’s Peak, CO (4,300 m) the following morning (HA *day 0*) where they resided for the next 22 days at the U.S. Army Research Institute of Environmental Medicine Maher Memorial Laboratory (*phase 2*; HA). During HA, participants were under constant supervision, consumed a controlled and measured diet, and engaged in prescribed physical activity. Diets contained either a standard or higher amount of protein, and were designed to induce weight loss, which is common during HA sojourn (33). The estimated energy deficit at HA was 70% or 1,849 kcal/day (SD 511) (9).

#### Study diets.

Beginning on HA1, the first full day of residence at 4,300 m, and continuing until they completed the HA phase of the study, participants consumed a controlled diet. Participants were randomized by study staff using computer-generated randomization to consume either a standard protein [SP; 1.1 g·kg^−1^·day^−1^ (SD 0.2)] or higher protein [HP; 2.1 g·kg^−1^·day^−1^ (SD 0.2)] diet during HA. Both diets were designed to provide 45% of energy as carbohydrate, while fat intake was reduced in HP to accommodate the higher protein intake (Table 1). Diets were primarily comprised of entrées, sides, and snack items included in U.S. military Meals Ready-to-Eat rations and were supplemented with fresh fruits and vegetables, fruit snacks, olive oil, and ranch-flavored salad dressing. A whey-protein beverage (Isopure Zero Carb; Isopure, Hauppauge, NY) was also provided as appropriate to manipulate protein intake. Water and noncaffeinated sodas were allowed ad libitum.

**Table 1.** Baseline participant characteristics, weight loss, and dietary intakes at sea level and over 22 days at high altitude

	SP		HP	
	SL	HA	SL	HA
Age, yr	23 ± 3		24 ± 7	
Body mass index, kg/m^2^	27.0 ± 4.0		25.5 ± 3.1	
Body fat, %	22.8 ± 7.0		22.6 ± 5.5	
V̇o_2peak_, ml·kg^−1^·min^−1^	49.2 ± 7.0		53.8 ± 7.2	
ΔWeight at HA, ‡ kg		−8.0 ± 2.6		−7.8 ± 1.2
ΔFFM at HA, ‡ kg		−4.0 ± 3.3		−3.2 ± 1.5
Energy intake, † kcal/day	2,366 ± 277	1,950 ± 186	2,418 ± 542	1,885 ± 269
Protein intake, %	16 ± 3	18 ± 2	14 ± 2	33 ± 2*
Protein intake, g/day	94 ± 20	88 ± 14	83 ± 15	154 ± 25*
Carbohydrate intake, %	50 ± 6	46 ± 1	54 ± 5	47 ± 1
Carbohydrate intake, g/day	300 ± 62	223 ± 24	327 ± 80	221 ± 35
Fat intake, %	34 ± 4	39 ± 2	32 ± 4	23 ± 3*
Fat intake, g/day	89 ± 9	84 ± 6	88 ± 7	47 ± 7*
Saturated fat intake, g/day	31 ± 4	26 ± 2	26 ± 7	15 ± 2*
Fiber intake, g/day	18 ± 7	21 ± 3	19 ± 10	18 ± 2*

Values are means ± SD; *n* = 8 from standard-protein diet group (SP) and 9 from higher-protein diet group (HP). SL, sea level (weight maintenance); FFM, fat-free mass; HA, high altitude (4,300 m; energy deficit). Adapted from Berryman et al. (9).

* Different from SP during HA, *P* < 0.05.

† For all dietary variables independent samples *t*-test were used to compare groups at SL (ad libitum diet) and HA (provided diet); no significant differences during SL.

‡ Linear mixed model, main effect of time, *P* < 0.001.

#### Questionnaires.

Modified versions of the Irritable Bowel Syndrome-Symptom Severity Score (IBS-SSS) Questionnaire (30) and the Gastrointestinal Quality of Life Index (GIQLI) Questionnaire (27) were administered weekly throughout the study. The GIQLI Questionnaire asked participants to subjectively rate the frequency of several GI-related symptoms (e.g., flatulence, constipation, loose stool, cramping, etc.), which were then used to compute an overall GIQLI score (27). AMS was assessed by Lake Louise score calculated from the shortened version of the Environmental Symptoms Questionnaire (6) and categorized as mild (≥0.7 and <1.53), moderate (≥1.53 and <2.63), and severe (≥2.63) (7).

#### Blood biochemistries.

Blood samples were collected by venipuncture and following a >10 h fast to measure markers of gut barrier function, stress, and inflammation. All samples were separated into serum or plasma and stored at −20 to −80°C until analysis. Plasma LPS-binding protein (LBP) was measured on SL *day 7* and HA *days 0*, *7*, and *21* by ELISA according to manufacturer’s instructions (Abonva, Taipei, Taiwan). Serum glucagon-like peptide-2 (GLP-2), a gastrointestinal hormone shown to modulate effects of diet-gut microbe interactions on GI permeability (14), was measured on SL *day 7* and HA *days 0*, *7*, and *21* by ELISA according to manufacturer’s instructions (EMD Millipore, St. Charles, MO). Serum IL-6 concentrations were measured on SL *day 0* and HA *days 0* and *19* by multiplex assay (Milliplex MAP; EMD Millipore). Serum cortisol concentrations were measured on SL *day 12* and HA *days 2*, *7*, *13*, and *19* by autoimmunoassay (Immulite 2000; Siemens Healthcare Diagnostics, Tarrytown, NY).

#### Small intestinal permeability.

Small intestinal permeability was assessed by quantifying the urinary excretion of orally ingested sugar substitutes (45, 53) on SL *day 12* and HA *days 1* and *18*. Measurements began in the morning with fasted participants consuming 5 g lactulose and 4 g mannitol dissolved in 180 ml of water. All urine produced over the next 5 h was collected. During the 5-h period, participants consumed the same individualized diet (breakfast and morning snack) on all 3 test days, with water allowed ad libitum. Urine aliquots were immediately frozen and stored at −20 to −80°C until analysis. Urine lactulose and mannitol concentrations were measured by HPLC with refractive index detection (Agilent 1100 HPLC, Santa Clara, CA) as previously described (51). Fractional excretion was calculated by multiplying the measured concentration of each probe by the total volume of urine collected and dividing by the dose administered. Small intestinal permeability was then calculated as the ratio of the fractional excretions of lactulose and mannitol (LM ratio) (45, 53).

#### Fecal sample collection and analysis.

Participants provided a single fecal sample during five separate time periods of the study; SL *days 0–4* (SL1) and *16–20* (SL2) and HA *days 1–2* (HA1), *8–11* (HA2), and *18–21* (HA3), to assess fecal microbiota composition and fecal concentrations of SCFAs, BCFAs, and ammonia. All samples were collected into plastic collection containers, immediately refrigerated, and processed within 3 h (SD 4; range: 5 min – 21 h) of production. Aliquots were immediately frozen and stored at −20 to −80°C until analysis.

#### Fecal metabolites.

Fecal SCFA and BCFA concentrations were measured as previously described (61, 87) with minor modifications. Fecal aliquots were thawed immediately before extraction, homogenized in distilled water (1:4 wt/vol), and centrifuged. Samples were then acidified using 50% H_2_SO_4_ (1:2 wt/vol), and fatty acids were extracted using diethyl ether (2:5 wt/vol). After incubating on ice for 2 min, samples were centrifuged, the organic layer was removed, and ethyl butyric acid was added as an internal standard. Samples were then stored at −80°C until analysis. Acetic acid, propionic acid, butyric acid, isobutyric acid, and isovaleric acid were quantified using an Agilent 7890A GC system with Flame Ionization Detection (60 m × 250 μm × 0.25 μm; DB-FFAP, Agilent J&W). Samples (1 μl) were injected by autosampler in triplicate using a split ratio of 10:1. The temperature program started with an initial temperature of 110°C for 2 min, increased 10°C/min up to 180°C, and was then maintained at 180°C for 5 min. The carrier gas was nitrogen with a constant flow of 1 ml/min. Calibration standards were included for each fatty acid, and used for peak identification and quantification.

Fecal ammonia concentrations were measured using a colorimetric assay according to manufacturer instructions (Abcam, Cambridge, MA).

#### Fecal microbiota composition.

DNA was extracted from fecal samples using the MoBio PowerFecal DNA isolation kit (Qiagen, Germantown, MD). Primers designed to amplify the V3-V4 region of the 16S rRNA gene were used for PCR amplification, and all samples were sequenced in triplicate on the Illumina MiSeq platform (Illumina, San Diego, CA). Sequencing data were processed using Quantitative Insights Into Microbial Ecology (QIIME) v.1.9.1 (16). Read quality assessment, filtering, barcode trimming, and chimera detection were performed on demultiplexed sequences using Trimmomatic (11). Reads were joined in QIIME using a minimum overlap of 32 bp and a maximum percent difference within the overlap of 20%. Operational taxonomic units (OTUs) were assigned by clustering sequence reads at 97% similarity and aligned against the Greengenes database core set v.13_8 (52) using PyNAST (15). Taxonomic assignment was completed using the RDP classifier v.2.2 (77).

Read counts averaged 80,024 reads/sample (SD 61,030; range: 31,267–417,621), and were grouped into 12,966 unique OTUs, which could be assigned to 134 unique genera and 13 unique phyla. For genus-level analyses, any OTUs that could not be assigned to a genus were grouped at the next lowest level of classification possible (e.g., family or order).

Diversity metrics were calculated after rarefaction at 31,267 reads/sample. Within-sample diversity (α-diversity) was calculated in QIIME using the Shannon and observed OTUs diversity metrics. Between-sample diversity (β-diversity) was measured using Bray-Curtis distances calculated using the R packages stats v.3.4.3 and phyloseq v.1.16.2. Ordinations of β-diversity metrics were then completed using principal coordinates analyses (PCoA) and average hierarchical clustering within the R package ape.

#### Statistical analysis.

Data were checked to verify adherence to model assumptions and transformed when necessary to meet model assumptions. Unless otherwise noted, statistical analyses were completed using SPSS v.21, data are presented as mean (SD), statistical significance was set at *P* ≤ 0.05, and *P* values between 0.05 and 0.10 were considered evidence of a trend for an effect.

All study outcomes except for genus-level read counts were analyzed by linear mixed models, generalized linear mixed models, or marginal models as appropriate (see table and figure legends for model specifications). All models accounted for the within-subject correlation and included diet group, time, and their interaction as fixed factors and age and baseline body mass index as covariates. The baseline value of the dependent variable was also included as a covariate in models where the dependent variable was measured at multiple SL time points (questionnaires and fecal outcomes) or if the first measurement at HA was completed immediately after ascent (plasma LBP and serum GLP-2). In all models, if a significant interaction or main effect was observed, post hoc comparisons were conducted using *t*-tests, and *P*-values were adjusted using Bonferroni corrections. Correlations were assessed using Pearson’s or Spearman’s correlation as appropriate or by including time-varying covariates in linear models.

Bray-Curtis dissimilarities in gut microbiota composition were analyzed using distance-based redundancy analysis in the R package vegan. Unrarefied genus-level read counts were analyzed using DESeq2 v.1.16.1 (47) and R v.3.4.2 to test for effects of diet, time, and their interaction on changes in gut microbiota composition while controlling for individual effects. For these analyses, likelihood ratio tests were used to test for diet-by-time interactions, and, if no interactions were observed, main effects of time. False discovery rate (FDR) was controlled by adjusting *P* values obtained for diet-by-time interactions and main effects of time using the Benjamini-Hochberg correction. If a significant interaction or main effect of time was observed (FDR ≤ 0.20), pairwise contrasts were examined for those taxa to identify between group differences or differences over time within the full cohort. *P*-values for pairwise contrasts were adjusted for the number of contrasts using Bonferroni corrections.

To address exploratory aims, participants were dichotomized as “responders” and “nonresponders” to the physiologic stress of HA using Lake Louise scores measured within the first 48 h of ascent (peak scores for all participants occurred within 48 h of ascent as expected; Ref. 7). Moderate or severe AMS (responder) was defined as a peak Lake Louise score of ≥1.53, and no or mild AMS (nonresponder) was defined as a peak Lake Louise score of <1.53 (6). The AMS-responder group was included in separate marginal or mixed models as an independent variable to examine changes in physiologic outcomes. All models accounted for the within-subject correlation and included diet group, time, and responder group as fixed factors, the responder group-by-time interaction, and age, baseline body mass index, and baseline value of the dependent variable as covariates. For analysis of changes in genus-level read counts over time, the AMS-responder group was substituted for diet group in the DESeq2 models.

Finally, linear discriminant analysis of effect size (LEfSe) (67) was used to identify taxa associated with AMS severity. For these analyses the AMS-responder group was used as the grouping factor (i.e., “class”), time point was used as a subgroup factor (i.e., “subclass”), and relative abundances of taxa measured at SL were included in the analysis. Default analysis parameters were used with the exceptions of reducing the significance threshold to *P* < 0.01 due to the small sample size and conducting pairwise between-group comparisons only within the same time points. As such, discriminant taxa were defined as those with an effect size >2.0 between groups (*P* < 0.01) at both SL time points.

## RESULTS

Primary and secondary results have been previously reported (9, 37, 50, 58, 84). Total body mass and fat-free mass losses during HA did not differ between diet groups (Table 1). During HA, protein intake was higher in HP relative to SP while fat intake was lower, and total fiber intake was marginally lower (Table 1).

### 

#### Effects of diet and time on GI symptoms and small intestinal permeability.

**Fig. 1.**
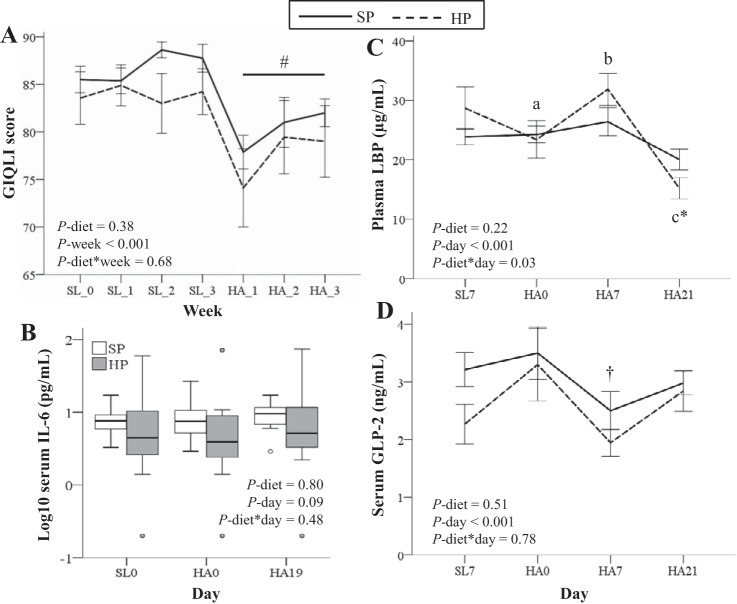
Changes in gastrointestinal (GI) symptomology, GI function, and inflammation at high altitude (HA; 4,300 m) are largely independent of dietary protein:fat ratio. GI quality of life (GIQLI; lower scores indicate worse symptoms; *A*) and fasting serum IL-6 (*B*), plasma LPS binding protein (LBP; *C*), and serum glucagon-like peptide (GLP)-2 (*D*) concentrations. Data are analyzed by marginal models controlling for age, body mass index, and baseline value. *B* and *C*: data were log_10_-transformed for analysis. HP, higher protein diet group; SL, sea level; SP, standard-protein diet group. HP, *n* = 9; SP, *n* = 8. Values are means ± SE. Time points not sharing a superscript letter are significantly different within a diet group (*P* ≤ 0.05). #Significantly different from SL (*P* < 0.05). *Significantly different from SP (*P*  = 0.02). †Significantly different from HA *days 0* and *21* (*P* ≤ 0.01).

GIQLI scores were lower (indicating worse symptomology) throughout HA relative to SL, and did not differ by diet group (Fig. 1*A*). IBS symptom severity scores did not differ over time (main effect of time, *P* = 0.69) or by diet group (diet-by-week interaction, *P* = 0.15) (data not shown).

Lactulose excretion was lower on HA *day 18* relative to SL and HA *day 1* while mannitol excretion was higher at SL relative to HA *days 1* and *18* (main effect of time, *P* ≤ 0.01, Table 2). The resulting LM ratio was 71% (SD 73) higher on HA *day 1* and 67% (SD 77) higher on HA *day 18* relative to SL (main effect of time, *P* < 0.001) indicating increased small intestinal permeability. Diet had no impact on the LM ratio (Table 2). When urine volume was included as a covariate in the model, the main effect of time for lactulose was no longer significant whereas results for mannitol excretion and the LM ratio were unchanged, the latter observation remaining consistent with increased small intestinal permeability at HA.

**Table 2.** Small intestinal permeability measured at sea level and high altitude

			*P* Value‡		
	SP	HP	Diet	Time	Diet × time
Lactulose,§ %			0.96	0.01	0.35
SL *day 1*2	0.15 ± 0.07	0.13 ± 0.04			
HA *day 1*	0.16 ± 0.10	0.15 ± 0.05			
HA day 18*	0.11 ± 0.10	0.12 ± 0.05			
Mannitol, %			0.41	<0.001	0.76
SL *day 1*2*	16.2 ± 4.4	17.6 ± 3.0			
HA *day 1*	10.5 ± 2.6	12.8 ± 5.1			
HA *day 18*†	8.6 ± 5.4	9.2 ± 2.6			
LM ratio§			0.47	<0.001	0.55
SL *day 1*2*	0.010 ± 0.005	0.007 ± 0.002			
HA *day 1*	0.014 ± 0.007	0.013 ± 0.005			
HA *day 18*	0.013 ± 0.007	0.013 ± 0.005			

Values are means ± SD; *n* = 8 from standard-protein diet group (SP) and 9 from higher-protein diet group (HP). SL, sea level (weight maintenance); HA, high altitude (4,300 m; energy deficit). LM, lactulose:mannitol.

* Significantly different from the other days (*P* < 0.05).

† Trend for significant difference relative to HA *day 1* (*P* = 0.08).

‡ Linear mixed model controlling for age and body mass index with Bonferroni corrections.

§ Log_10_ transformed for analysis.

Diet had no significant effect on serum IL-6 concentrations; however, an upwards trend was observed over time at HA (Fig. 1*B*). Changes in plasma LBP concentrations over time differed by diet with concentrations initially increasing at HA in HP but then dropping to a concentration 24% lower than that measured in SP on HA *day 21* (*P* = 0.02; Fig. 1*C*). Diet had no effect on serum GLP-2 concentrations (Fig. 1*D*). However, in the full cohort, GLP-2 concentrations were lower on HA *day 7* relative to HA *days 0* and *21* and were inversely correlated with LBP concentrations over time [β: −2.3 µg/ml (SE 0.9), *P* = 0.01].

#### Effects of diet and time on fecal metabolite concentrations.

Total fecal SCFA concentrations were 39% lower during the second week of HA in HP relative to SP due to lower acetate, propionate and butyrate concentrations in HP but did not differ before or after (Table 3). Total BCFA concentrations trended toward being higher in SP relative to HP independent of diet (Table 3) due to higher mean concentrations of isovalerate (main effect of diet, *P* = 0.07) in SP throughout the study (data not shown). Fecal ammonia concentrations did not differ by diet but were higher during the second and third weeks at HA relative to SL (Table 3).

**Table 3.** Fecal short-chain fatty acid, branched-chain fatty acid, and ammonia concentrations measured at sea level and high altitude

			*P* Value^a^		
	SP	HP	Diet	Time	Diet × time
Acetate, µmol/g wet wt			0.42	0.18	0.08
SL1	35.0 ± 12.1	29.7 ± 14.9			
SL2	37.1 ± 26.5	24.5 ± 8.6			
HA1	26.2 ± 6.9	23.7 ± 6.7			
HA2	28.2 ± 8.9	18.6 ± 10.0^b^			
HA3	20.2 ± 8.7	22.3 ± 2.3			
Propionate, µmol/g wet wt			0.79	0.14	0.03
SL1	15.4 ± 8.1	16.9 ± 9.5			
SL2	14.7 ± 12.8	13.6 ± 5.2			
HA1	12.5 ± 4.9	10.1 ± 4.1			
HA2	14.1 ± 7.9	8.3 ± 4.5^b^			
HA3	7.2 ± 3.7^c^	10.6 ± 3.5			
Butyrate, µmol/g wet wt			0.29	0.07	0.02
SL1	13.6 ± 6.2	13.6 ± 9.2			
SL2	14.1 ± 12.2	9.7 ± 4.2			
HA1	13.0 ± 3.9	11.7 ± 2.3			
HA2	12.8 ± 4.2	6.7 ± 4.8^b^			
HA3	10.4 ± 4.7	10.9 ± 3.4			
Total SCFA, µmol/g wet wt			0.55	0.19	0.04
SL1	64.1 ± 23.8	60.2 ± 30.3			
SL2	66.0 ± 51.0	47.8 ± 16.2			
HA1	51.6 ± 13.4	45.6 ± 9.5			
HA2	55.1 ± 19.3	33.6 ± 18.7^b^			
HA3	37.9 ± 15.2	43.8 ± 6.2			
BCFA, µmol/g wet wt			0.07	0.32	0.29
SL1	3.2 ± 2.2	2.6 ± 1.4			
SL2	3.1 ± 2.7	1.6 ± 1.6			
HA1	3.7 ± 2.9	2.2 ± 1.9			
HA2	3.8 ± 1.7	1.9 ± 1.0			
HA3	1.8 ± 1.2	2.1 ± 1.8			
Ammonia, µmol/g wet wt			0.38	0.001	0.42
SL1	38.0 ± 23.9	24.2 ± 17.9			
SL2	16.1 ± 7.4	18.6 ± 12.3			
HA1	34.2 ± 21.1	23.9 ± 15.1			
HA2^d^	59.1 ± 27.3	29.5 ± 15.5			
HA3^e^	28.9 ± 14.1	28.4 ± 17.0			

Values are means ± SD; *n* = 8 from standard-protein diet group (SP) and 9 from higher protein diet group (HP). SL, sea level (weight maintenance); HA, high altitude (4,300 m; energy deficit); BCFA, branched chain fatty acid (isobutyrate and isovalerate); SCFA, short-chain fatty acid (acetate, propionate, and butyrate). Samples collected on study *days 0–4* (SL1) and *16–20* (SL2) at sea level and *days 1–2* (HA1), *8–11* (HA2), and *18–21* (HA3) at high altitude.

a Linear mixed model with Bonferroni corrections controlling for age, body mass index, and concentration on SL day 0–4. All concentrations were log_10_- transformed for analysis.

b Significantly different from SP (*P* ≤ 0.04).

c Significantly different from HA2 within SP (*P* = 0.05).

d Significantly different from SL2 (*P* < 0.001).

e Trend for difference from SL2 (*P* = 0.06).

#### Effects of diet and time on fecal microbiota composition.

**Fig. 2.**
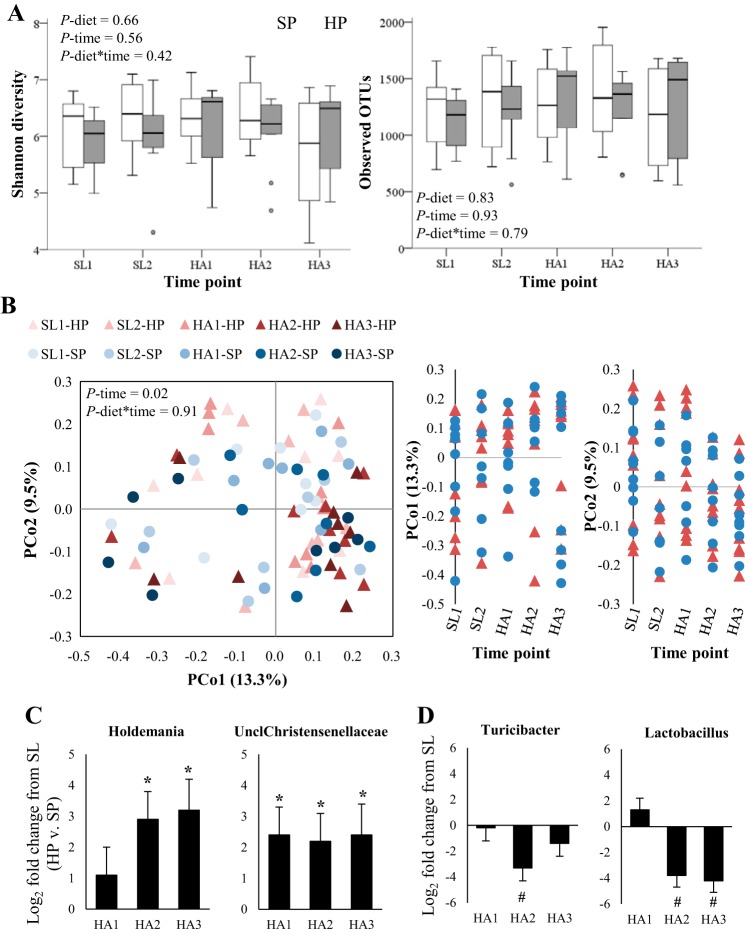
Changes in fecal microbiota composition during weight loss at high altitude (HA 4,300 m) are largely independent of dietary protein:fat ratio. *A*: α-diversity [Shannon diversity and observed operational taxonomic units (OTUs)] analyzed by marginal models controlling for age, body mass index, and baseline diversity (i.e., SL1). *B*: principle coordinates (PCo) analysis of Bray-Curtis dissimilarities. Individual data points represent the entire fecal microbiota community of a single individual at one point in time. Samples closer together are more similar than samples farther apart. *C*: log_2_-fold change in relative abundance from SL in HP relative to SP for taxa demonstrating significant diet-by-time interactions (false discovery rate <0.20). *Change from SL significantly different in HP vs. SP (*P* ≤ 0.02). *D*: log_2_-fold change in relative abundance from SL in genera demonstrating a significant main effect of time (false discovery rate <0.20). #Significant change from SL (*P* ≤ 0.005). HA, high altitude; HP, higher protein diet group; SL, sea level; SP, standard protein diet group; Uncl, unassigned genus level taxonomy. HP, *n* = 9; SP, *n* = 8.

At the community level, no metric of α-diversity changed over time or differed by diet (Fig. 2*A*). PCoA (Fig. 2*B*) and hierarchical clustering (data not shown) of Bray-Curtis dissimilarities did not reveal any clustering by diet (diet-by-time interaction, *P* = 0.91). The effects of diet on genus-level fecal microbiota relative abundances were observed for only two taxa. Both changes in the relative abundances of *Holdemania* (phyla: Firmicutes) and unclassified taxa within the *Christensenellaceae* family (phyla: Firmicutes) at HA were greater in HP relative to SP (diet-by-time interaction, *P* ≤ 0.003; FDR < 0.20; Fig. 2*C*). However, both taxa demonstrated a trend toward being less abundant in HP relative to SP at SL (LEfSe effect size ≤ 2.6, *P* < 0.02), which implicated regression to the mean rather than an effect of diet. Finally, no between group difference was noted when LEfSe was used to examine differences in fecal microbiota composition measured during the final week of HA (*P* > 0.01; data not shown). Taken together these findings indicated that diet had little impact on fecal microbiota community composition.

PCoA of Bray-Curtis dissimilarities did show evidence of clustering over time (Fig. 2*B*, main effect of time, *P* = 0.02). In support, several genera demonstrated significant changes over time independent of diet (main effect of time, FDR <0.20). Specifically, relative to SL, *Lachnospira* relative abundance was lower on HA days 1–2 [log_2_ fold change = −1.5 (SE 0.5); *P* = 0.005] but not thereafter, *Turicibacter* relative abundance was lower during only the second week at HA (Fig. 2*D*), and *Bacteroides* relative abundance trended toward being higher during only the second week at HA [log_2_ fold change = 0.9 (SE 0.4); *P* = 0.06]. *Lactococcus, Streptococcus*, and *Lactobacillus* (Fig. 2*D*) relative abundances were all lower relative to SL during the second and third weeks of HA (range of log_2_ fold changes = 2.2–4.9; *P* ≤ 0.005). Of note, all three of these taxa include strains that are commonly used as starter cultures in dairy products (*Streptococcus thermophilus* and *Lactobacillus* spp. in yogurt and *Lactococcus* spp. in cheese). Therefore, SL food records were used to estimate yogurt consumption during SL (consumption of *Lactococcus-*containing cheeses during SL could not be accurately estimated). Yogurt consumption during SL was correlated with mean *Streptococcus* relative abundance during SL (Spearman’s ρ = 0.68, *P* = 0.003) and the change in *Streptococcus* relative abundance from SL to the third week at HA (Spearman’s ρ = −0.71, *P* = 0.002). In contrast, SL yogurt consumption was not correlated with mean *Lactobacillus* relative abundance during SL (Spearman’s ρ = 0.08, *P* = 0.75) or the change in *Lactobacillus* relative abundance from SL to the third week at HA (Spearman’s ρ = −0.03, *P* = 0.89). These observations suggest that the decrease in *Streptococcus*, but not *Lactobacillus*, relative abundance at HA was attributable to reduced yogurt consumption. Of note, neither mean dietary fiber intake at SL nor the change in mean fiber intake from SL to HA was correlated with changes in the relative abundance of any of these six taxa (*P* ≥ 0.24 for all).

#### Responders and nonresponders: AMS severity.

AMS severity as measured by the Lake Louise score represents an overall cognitive and physical response phenotype resulting from the physiologic stress induced by hypobaric hypoxia. Therefore, in additional exploratory analyses, AMS severity was considered an indicator of the cumulative stress response to HA, and associations between gut microbiota-related factors and both the development of and response to AMS were explored.

**Fig. 3.**
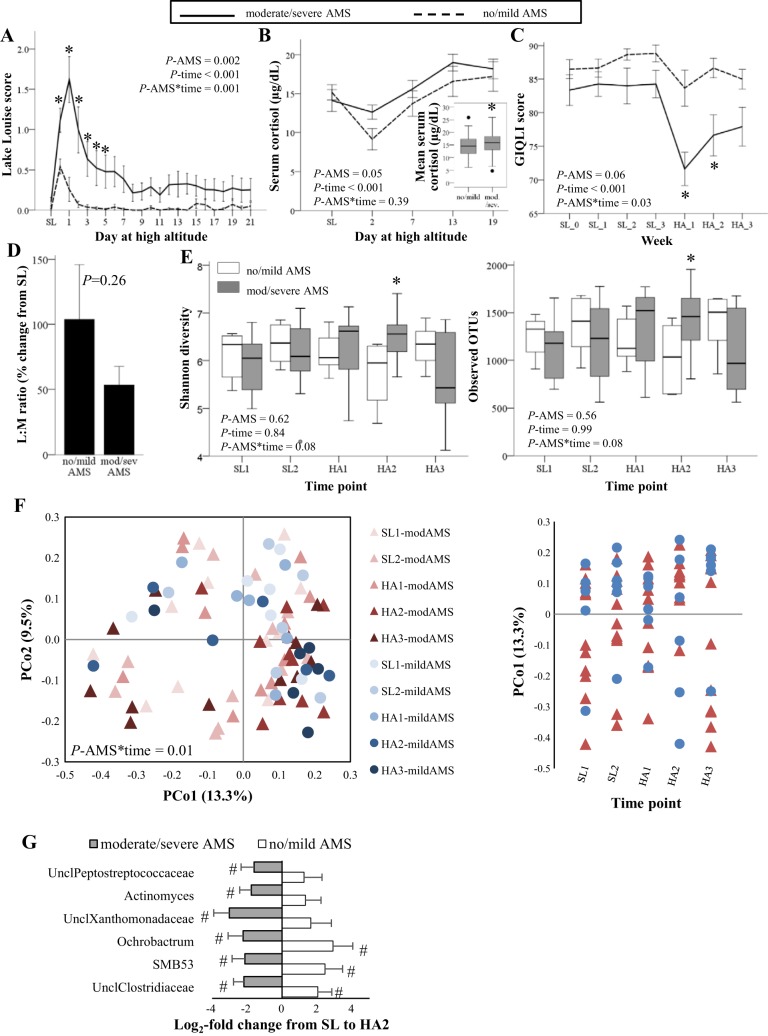
Acute mountain sickness (AMS) severity is associated with gastrointestinal (GI) symptoms, and changes in fecal microbiota composition. Lake Louise scores (*A*), serum cortisol (*B*), GI symptomology (*C*), lactulose:mannitol ratio (L:M; *D*), and α-diversity (*E*) by AMS severity group (no/mild vs. moderate/severe). *A*–*D*: data are means ± SE and analyzed by marginal models or mixed models controlling for diet group, age, body mass index, and baseline value. *Different from no/mild AMS (*P* < 0.05). *F*: principle coordinates analysis of Bray-Curtis dissimilarities. Individual data points represent the entire fecal microbiota community of a single individual at one point in time. Samples closer together are more similar than samples farther apart. *G*: log_2_-fold change in relative abundance from SL to HA2 in taxa demonstrating significant AMS group-by-time interactions (false discovery rate <0.20). #Significant within group difference from SL. HA, high altitude; SL, sea level; Uncl, unassigned genus level taxonomy. No/mild AMS, *n* = 6; moderate/severe AMS, *n* = 11.

Eleven participants reported moderate or severe AMS (responders) at ≥1 time points during the first 48 h at HA (Table 4). Lake Louise scores were consistently higher during the first 6 days of sojourn in AMS responders relative to those who experienced no or mild AMS (nonresponders) (Fig. 3*A*). In support of AMS severity reflecting the cumulative stress response, mean serum cortisol concentrations were slightly higher throughout HA in AMS responders relative to nonresponders (Fig. 3*B*). Finally, GIQLI scores were lower during the first 2 wk of sojourn in AMS responders relative to nonresponders (Fig. 3*C*), consistent with GI issues contributing to AMS symptomology. No differences in baseline characteristics between responder groups were noted (Table 4) nor were any relationships observed with changes in the LM ratio (Fig. 3*D*), plasma LBP concentrations (data not shown), or fecal metabolite concentrations (data not shown). Lake Louise scores were higher in SP relative to HP on HA *day 0* [before the prescribed diets were administered; mean difference = 0.6 (95% confidence interval: 0.2, 0.9), *P* = 0.004] and on HA *day 2* [mean difference = 0.7 (95% confidence interval: 0.3, 1.1), *P* = 0.001] but did not differ other days (diet-by-day interaction, *P* = 0.02). In contrast, AMS severity did not differ between diet groups on any day (diet-by-day interaction, *P* = 1.0), and frequency of AMS responder/nonresponder classification did not differ by diet group (Table 4), indicating that the incidence of moderate-to-severe AMS was not affected by dietary protein:fat ratio.

**Table 4.** Participant characteristics categorized by peak acute mountain sickness severity measured within 48 h of ascent to high altitude

	AMS Severity		
	None or mild	Moderate or severe	*P* value
SP/HP, *n*	3/3	5/6	0.86
Age, yr	23 ± 4	24 ± 6	1.00
Body mass index, kg/m^2^	24.7 ± 3.7	27.0 ± 3.3	0.20
Body fat, %	20.6 ± 6.7	23.8 ± 5.7	0.30
V̇o_2peak_, ml·kg^−1^·min^−1^	51.8 ± 6.3	51.5 ± 8.1	0.96
LM ratio	0.009 [0.005]	0.008 [0.004]	0.72
α-Diversity			
Shannon	6.2 ± 0.5	6.0 ± 0.7	0.32
Observed OTU	1,313 ± 269	1,154 ± 364	0.15
*Bacteroides:Prevotella* ratio	234 [2,219]	1 [37]	0.07

Data were measured at sea level and are means ± SD or median [interquartile range]. Frequencies were measured using χ^2^-tests, and means by independent samples *t*-test. Marginal models were used to analyze gut microbiota metrics, which include 2 measurements at sea level. Acute mountain sickness was measured by Lake Louise score. HP, higher protein diet group; LM ratio, lactulose:mannitol ratio measurement of small intestinal permeability; OTU, operational taxonomic unit; SP, standard-protein diet group; SL, sea level (weight maintenance); HA, high altitude (4,300 m; energy deficit).

Differences in fecal microbiota composition between AMS responder groups were observed before ascent to HA. Specifically, LEfSe analyses of SL gut microbiota composition indicated that *Prevotella* (phyla: Bacteroidetes) relative abundance at SL was higher in AMS responders relative to nonresponders (effect size = 4.2, *P* = 0.006). Furthermore, the *Bacteroides:Prevotella* ratio, which is known to drive interindividual variation in human gut microbiota composition (18), trended toward being lower at SL in the AMS responders (Table 4). We previously reported that the proportion of total body mass loss at HA attributable to fat-free mass also demonstrated substantial interindividual variability in this cohort with 7 participants losing predominantly fat-free mass and 10 losing predominantly fat mass (9). Interestingly, the mean *Bacteroides:Prevotella* ratio at SL was lower in individuals whose total body mass loss at HA was >50% fat-free mass relative to those whose total body mass loss at HA was >50% fat mass [median (interquartile range): 1 (1) vs. 203 (1,317), *P* = 0.01]. *Prevotella* was also the most discriminant taxa between these two groups, being enriched in the SL microbiota of individuals whose weight loss at HA comprised >50% fat-free mass (LEfSe effect size = 4.4, *P* = 0.0001).

Differences in fecal microbiota composition between AMS responder groups were also observed after ascent to HA. A trend for an AMS responder group-by-time interaction (*P* = 0.08) was observed for both the Shannon and observed OTUs α-diversity metrics (Fig. 3*E*). Post hoc testing indicated differences were present only during the second week at HA during which Shannon diversity was 15% higher and observed OTUs were 45% higher in AMS responders relative to nonresponders (*P* = 0.03 for both). These results were unchanged after excluding two individuals from the moderate/severe AMS group who reported constipation during HA2, and bowel movement frequency during HA2 did not differ between AMS responder groups [median: nonresponder = 5 per week (interquartile range 8) vs. responder = 5 per week (interquartile range 5), *P* = 0.56]. These observations indicated that increased GI transit time, which has been associated with greater α-diversity (63), likely did not explain between group differences. Furthermore, Bray-Curtis dissimilarities demonstrated clustering over time as a function of AMS responder group (Fig. 3*F*; group-by-time interaction, *P* = 0.01). Responder group-by-time interactions (FDR <0.20) were also observed for six taxa, which were all within the top third of the most abundant taxa, and all decreased in relative abundance from SL to HA2 in AMS responders relative to nonresponders (Fig. 3*G*).

## DISCUSSION

The first aim of this study was to determine the effect of increasing the dietary protein:fat ratio on gut microbiota composition, gut microbiota-derived metabolites, GI barrier function, and GI symptoms during weight loss at HA. Results demonstrated that protein:fat ratio had little impact on fecal microbiota composition and only transient effects on fecal SCFA and plasma LBP concentrations. These effects did not appear clinically meaningful as no between-group differences in intestinal permeability, GI symptoms, incidence of moderate-to-severe AMS, or inflammation were observed. Although previous studies have reported unfavorable effects of higher protein diets on fecal microbiota composition and metabolites, those results were more likely attributable to reductions in fiber intake because the taxa most consistently affected were saccharolytic (12, 19, 24, 25, 65) and decreased in proportion to reductions in fiber intake (24, 25, 65). Our findings are more consistent with experiments conducted under normal environmental conditions in which dietary macronutrient composition was manipulated within recommended intake ranges and fiber intake was matched between groups (5, 79). Those studies also did not demonstrate detrimental effects of higher protein diets on markers of GI health despite evidence of increased protein fermentation and/or decreased carbohydrate fermentation (5, 8, 79). As such, any impact of gut microbes and their metabolites on GI barrier function at HA is likely to be independent of dietary protein:fat ratio within the macronutrient intake ranges and time period studied.

The second aim of this study was to identify associations among the gut microbiota, weight loss at HA, and the host response to HA as measured by AMS severity. Notably, AMS severity and weight loss at HA were not affected by diet but demonstrated associations with fecal microbiota composition and, in the case of AMS severity, GI symptoms, providing preliminary evidence of a possible role for the gut microbiota in individual responses to HA.

Recent evidence indicates that the gut microbiota warrants consideration in studies aiming to explain interindividual variance in human phenotypes in response to different exposures (43, 85), and it has been established that the *Bacteroides:Prevotella* ratio is a predominant driver of interindividual variability within the human gut microbiota (18). Intriguingly, AMS severity, a phenotype known to demonstrate large unexplained interindividual variability (7), and body composition changes during weight loss at HA, which also demonstrated large interindividual variability (9), were both associated with an enrichment of *Prevotella* at SL and a correspondingly lower *Bacteroides:Prevotella* ratio. These results suggest that the ratio of *Bacteroides* to *Prevotella* in the gut microbiota, a well-established driver of interindividual variability in human gut microbiota composition, may contribute to the interindividual variability in host responses at HA.

That higher *Prevotella* abundance was associated with worse AMS symptomology and greater fat-free mass losses at HA was somewhat unexpected. A higher ratio of *Prevotella* to *Bacteroides* is generally considered a marker of a healthy high-fiber, plant-rich diet (18, 32, 80), *Prevotella* are known SCFA producers (48), and certain *Prevotella* species have been associated with improved glucose homeostasis (43), which could be beneficial for mitigating impairments in glucose tolerance common during HA sojourn (84). However, *Prevotella* can also be detrimental depending on the environment (44). For example, recent studies suggest that *Prevotella* may thrive during oxidative stress, promoting intestinal mucus barrier dysfunction and inflammation (26, 66), and some species can act as opportunistic pathogens (29). *Prevotella*-dominated microbiota are also specialized in the degradation of plant fibers and have decreased lipolytic and proteolytic fermentation potential (76). This could have altered the availability of substrates from the low plant-fiber diets used in the present study thereby impacting body composition. Of note, the *Bacteroides:Prevotella* ratio has been correlated with long-term habitual dietary patterns but appears to be relatively stable over short time frames, even in response to substantial dietary changes (18, 80). As such, to what extent the *Bacteroides:Prevotella* ratio provides a useful intervention target for improving host responses to HA is unclear, especially as current knowledge suggests that diets designed to increase the *Bacteroides:Prevotella* ratio would likely be high in animal fat and protein and low in fiber (80). Rather, these preliminary findings suggest that the *Bacteroides:Prevotella* ratio could serve as a possible marker for identifying individuals who may be more susceptible to the effects of HA.

AMS severity was also associated with higher fecal microbiota diversity a week after AMS onset. Of note, our group recently documented an increase in gut microbiota diversity in association with larger increases in GI permeability in soldiers engaged in a multiple-stressor military training exercise conducted at low altitude (39). These observations, which correlate increased diversity with worse responses to stress, contrast with animal studies reporting decreased microbiota diversity following exposure to various psychological and physiologic stressors (10, 71, 72). The discrepancy could reflect differential impacts of separate stressors on the gut microbiota (38) and/or the recognized difficulty in extrapolating animal microbiota studies to humans (56). Interpreting our findings is also complicated by an inability to separate cause and effect. For example, as higher diversity is generally considered a marker of a healthy and resilient microbiota (62), the increase in diversity in individuals with a more severe stress response observed in our human studies could reflect a beneficial adaptation to stress. This hypothesis would be consistent with murine studies of cold stress in which changes in the gut microbiota facilitate host acclimatization to the cold (17, 89). Alternately, our observations may simply reflect greater, transient changes in the colonic environment in individuals with a more severe stress response. Future studies are needed to separate cause and effect in the relationship between the human gut microbiota and host stress response, while also determining the extent to which any stress-induced restructuring of the gut microbiota is sustained.

Independent of AMS symptomology, weight loss at HA was associated with reduced relative abundance of both *Lactobacillus* and *Turicibacter*. The observed decrease in *Lactobacillus* relative abundance at HA was of particular interest as this SCFA-producing genus promotes GI barrier integrity, immune function, and resistance against enteric pathogens (31). The transient decrease in *Turicibacter* relative abundance during HA was also notable as *Turicibacter* depletion has been reported in rodent models of immunodeficiency (20, 40) and inflammatory bowel disease (3, 64) in association with increased inflammation (46). Our findings therefore raise the possibility that the physiologic effects of weight loss at HA may include *Lactobacillus* and *Turicibacter* depletion, which could in turn contribute to reduced GI barrier integrity, inflammation, altered immune function, and heightened susceptibility to bacterial pathogens, which have been reported at HA (41, 54). On the other hand, previous studies have not reported a reduction in *Lactobacillus* during HA sojourn (*Turicibacter* was not measured) (2, 42) or reductions in the relative abundances of these taxa in individuals exposed to normobaric hypoxia simulating ~4,000-m altitude (68–70). The latter observation implies that sustained hypoxia per se is likely not the mechanism underpinning observed changes in relative abundance and that some combination of hypobaria, hypoxia, weight loss, increased physical activity, and dietary change is responsible. Of note, all of these factors are inevitable or common during HA sojourn (33).

This combination of factors was also associated with a physiologic stress response and increased inflammation, which is consistent with previous reports (34, 41), and could drive changes in gut microbiota composition. In addition, a rapid and sustained increase in small intestinal permeability was observed. To our knowledge, only one previous study has directly measured intestinal permeability during HA sojourn. In that observational study of mountaineers, Dinmore et al. (21) reported increased small intestinal permeability at 5,570 m but attributed the response to residual effects of GI infection. However, rodent studies have shown that intestinal permeability increases upon exposure to hypobaric hypoxia (82, 88), attributing the response to inflammation, oxidative stress, atrophy, and villous collapse within the GI epithelium (1, 82, 86, 88). Although the present study did not directly address those mechanisms, the progressive decrease in mannitol excretion is consistent with the findings of Dinmore et al. (21) and suggests that small intestinal absorptive capacity and/or surface area was reduced during weight loss at HA, which could reflect villous atrophy.

Our data suggest that the pleiotropic hormone GLP-2 could be one factor impacting GI permeability at HA. GLP-2 is secreted from intestinal enteroendocrine cells and is thought to benefit GI function in part by stimulating blood flow, reducing inflammation, and enhancing GI barrier integrity (14, 22, 23). Furthermore, elevated GLP-2 concentrations following intestinal injury and in response to fasting have been shown to facilitate GI growth and repair (22). We hypothesize that a reduction in enteroendocrine cell density resulting from diminished intestinal surface area, as suggested by the decrease in mannitol excretion, could underpin the observed transient decrease in GLP-2 concentrations, which in turn could contribute to an increase in intestinal permeability (14). The subsequent GLP-2 rebound may reflect an adaptive response to intestinal injury and underfeeding to promote GI growth and repair. The inverse correlation with plasma LBP further implicates GLP-2 as having influenced GI barrier function, although the sustained increase in intestinal permeability indicates that other unmeasured factors also contributed.

Study findings should be interpreted within the context of several limitations. First, the sample size was small, and some between-group differences were likely not detected. As such, the absence of effects of diet composition on many study outcomes warrants cautious interpretation and requires replication. Second, the absence of a weight-maintenance control group at HA precludes determining whether temporal changes in study outcomes were attributable to hypobaric hypoxia and acclimatization, weight loss, changes in diet, other factors, or their combination. Third, study outcomes were not all assessed at the same time points which prevented examination of or complicated interpretation of associations between outcomes. Fourth, functional assessment of GI permeability was limited to the small intestine, and it cannot be assumed that small intestinal permeability mirrors that of the large intestine where gut microbes and their metabolites are more abundant. Additionally, intestinal permeability was measured only once at SL which prevented assessing whether any stress related to research study participation was associated with an increase in intestinal permeability at SL. However, even if such an effect were present, it did not mask the increases in permeability observed at HA. Finally, the findings related to weight loss at HA, AMS severity, and the gut microbiota must be considered preliminary and hypothesis generating due to the small sample size and exploratory and correlative nature of the analysis.

In summary, this tightly controlled study integrated stress-induced changes in physiology with temporal changes in the gut microbiota and its metabolites, and objective and subjective measures of GI health and function thereby allowing for an analysis of multiple steps along the pathway linking environment, stress, the gut microbiota, and host health. Findings demonstrated several novel associations between the gut microbiota and host responses to HA that were independent of dietary protein:fat ratio. Intriguingly, the ratio of *Bacteroides* to *Prevotella* in the gut microbiota, a well-established driver of interindividual variability in human gut microbiota composition, was associated with interindividual variability in host responses at HA, and increased gut microbiota diversity, considered a marker of a healthy and resilient microbiota, was elevated after HA exposure in individuals who experienced a more severe stress response at HA as measured by AMS symptomology. Although the study design precluded establishing a causal role for the gut microbiota in host responses to HA, findings provide preliminary evidence for a potential role, and support the need for additional research designed to determine if the gut microbiota can be leveraged to improve physiologic responses to HA.

## GRANTS

This work was supported by the U.S. Army Medical Research and Materiel Command and the U.S. Department of Defense, Defense Health Program.

## DISCLAIMERS

The opinions or assertions contained herein are the private views of the authors and are not to be construed as official or reflecting the views of the Army or the Department of Defense. Any citations of commercial organizations and trade names in this report do not constitute an official Department of the Army endorsement or approval of the products or services of these organizations. Approved for public release; distribution is unlimited.

## DISCLOSURES

No conflicts of interest, financial or otherwise, are declared by the authors.

## AUTHOR CONTRIBUTIONS

J.P.K., C.E.B., A.J.Y., J.C.R., and S.M.P. conceived and designed research; J.P.K., C.E.B., A.J.Y., P.N.R., and S.M.P. performed experiments; J.P.K., C.E.B., T.A.B., and I.G.P.-F. analyzed data; J.P.K., C.E.B., A.J.Y., I.G.P.-F., and S.M.P. interpreted results of experiments; J.P.K. prepared figures; J.P.K. drafted manuscript; J.P.K., C.E.B., A.J.Y., P.N.R., T.A.B., I.G.P.-F., J.C.R., and S.M.P. edited and revised manuscript; J.P.K., C.E.B., A.J.Y., P.N.R., T.A.B., I.G.P.-F., J.C.R., and S.M.P. approved final version of manuscript.
